# The Performance of Graphene-Enhanced THz Grating: Impact of the Gold Layer Imperfectness

**DOI:** 10.3390/ma15030786

**Published:** 2022-01-20

**Authors:** Patrizia Lamberti, Monica La Mura, Vincenzo Tucci, Erick Nkyalu, Ali Khan, Marina Yakovleva, Nadzeya Valynets, Alesia Paddubskaya, Aleksandr Saushin, Viatcheslav Vanyukov, Marian Baah, Andrzej Urbanowicz, Yuri Svirko, Polina Kuzhir

**Affiliations:** 1 Department of Information and Electrical Engineering and Applied Mathematics, University of Salerno, 84084 Fisciano, SA, Italy; mlamura@unisa.it (M.L.M.); vtucci@unisa.it (V.T.); 2 Department of Physics and Mathematics, Institute of Photonics, University of Eastern Finland, 80100 Joensuu, Finland; erickn@student.uef.fi (E.N.); alikhan@student.uef.fi (A.K.); aleksandr.saushin@uef.fi (A.S.); viatcheslav.vanyukov@uef.fi (V.V.); marian.baah@uef.fi (M.B.); yuri.svirko@uef.fi (Y.S.); polina.kuzhir@uef.fi (P.K.); 3 Institute for Nuclear Problems, Belarusian State University, 220030 Minsk, Belarus; yakovlevmarin@gmail.com (M.Y.); Nadezhda.Volynets@gmail.com (N.V.); paddubskaya@gmail.com (A.P.); 4 CNRS, Centre de Nanosciences et de Nanotechnologies, Université Paris-Saclay, 91120 Palaiseau, France; 5 Udmurt Federal Research Center of the UB RAS, Institute of Mechanics, 426067 Izhevsk, Russia; 6 Department of Optoelectronics, Center for Physical Sciences and Technology, 02300 Vilnius, Lithuania; andzej.urbanovic@ftmc.lt; 7 UAB “TERAVIL”, 02300 Vilnius, Lithuania

**Keywords:** Drude–Smith model, Fabry–Pérot resonances, FEM model, gold non-ideality, graphene, multilayer device, THz time domain spectroscopy

## Abstract

We report the performance of a graphene-enhanced THz grating fabricated by depositing a gold layer on the femtosecond micromachined SiO_2_ substrate. The morphology of the gold plated patterned substrate was studied by scanning electron microscopy (SEM) and atomic force microscopy (AFM), while the quality of the chemical vapor deposition (CVD) graphene was evaluated by Raman spectroscopy. The electromagnetic (EM) response of the metasurface comprising the graphene sheet and the gold plated substrate was studied by THz time domain spectroscopy in the 100 GHz–1 THz frequency range. We employed the finite elements method (FEM) to model the metasurface EM response by adjusting the ac conductivity of the gold layer covering the patterned SiO_2_ substrate to reproduce the measured transmission/reflection spectra. The results of the numerical simulation reveal the impact of the imperfectness of the gold layer on the performance of the THz metasurface. The experimental results are well described in terms of the Drude–Smith model of metal conductivity that takes into account the anisotropic scattering of the carriers in thin metal films.

## 1. Introduction

The concept of metamaterials has brought a lot of attention [1,2,3,4,5] to two- and three-dimensional subwavelength structures capable of manipulating electromagnetic (EM) radiation. The metamaterials’ properties are determined by the material of meta-atoms, their mutual arrangement, and collective resonance modes arising from their EM coupling. Being fabricated by electron beam lithography, nanoimprinting [6], self-organization [7], or 3D printing [8], metamaterials are reproducible and technology friendly. Layered [9] or 3D structured [10] architectures capable of matching the free space impedance [11] allow one to fabricate passive EM devices such as filters, lenses, collimators, polarizers, attenuators, perfect absorbers, and bolometers, to name a few. Introducing graphene makes it possible to achieve perfect and—even more important—tunable THz absorption [12,13].

This is because graphene, a one atom thick graphite layer, absorbs 2.3% of light in optical frequencies owing to interband transitions [14]. At lower frequencies, predominating intraband transitions [15] lead to even 50% absorptance for the free-standing graphene film [16]. By placing graphene on the top of the quarter wavelength thick dielectric slab matching free space and graphene admittances [12], one can achieve absorptance as high as 95%, i.e., approaching a perfect absorber at a particular wavelength. On the other hand, for free-standing graphene, the 50% absorptance condition is wavelength-independent and can be achieved when the admittance of free space is equal to half of the sheet conductance of graphene (or graphene multilayer). Thereby, being easily tunable via applying any external forces (mechanical deformation [17], biasing [18], and laser irradiation [13]) and non-dispersive in a wide frequency range, i.e., 100 GHz–1.5 THz [19], graphene proves to be a unique material basis to design stable and tunable absorptive EM devices.

Here, we consider the metasurface composed of conventional gold-plated SiO_2_ diffraction grating enhanced with graphene. Especially, the grating has the specific effect of diffracting the incident wave rather than simply reflecting it; in this way, the energy of the radiating source spreads along different directions. Consequently, multiple reflections are triggered between the grating cavities and the graphene layer, thus improving the overall absorption. The evanescent modes, excited owing to the diffraction of the incident wave on the grating, enable an increase in the absorption in the graphene sheet. Although the considered device is very simple, it provides an efficient playground to visualize the robustness of the graphene-enhanced device in the presence of the defects in graphene and/or metal layers and the imperfectness of the patterned dielectric lattice.

It has been demonstrated [20] that EM properties of graphene in the THz range remain unaffected if defects occupy 10% or less of its area. However, when a graphene sheet is employed as an element of the metasurface, the performance of the whole structure also depends on the imperfectness of the patterned SiO_2_ substrate, which may affect the uniformity of the gold layer and prevent achieving the perfect absorption condition.

It is worth mentioning that metallic reflection requires at least the skin depth thick metal layer. In the THz range (wavelength is 300 μm at 1 THz), this implies deposition of the gold layer thicker than 30 nm. The conductivity of such a nanometrically thin film may be strongly influenced by its integrity and homogeneity, e.g., the density of the voids and pores, granule structure, the presence of micro-cracks, and the dislocations caused by an irregular substrate surface. These defects, which lead to a lower ac surface conductivity in comparison with that of the “bulk” gold, often occur in the films fabricated by conventional magnetron sputtering and thermal evaporation [21], and are even more pronounced in films deposited on top of rough surfaces. The deviation from the Drude-like behavior of the conductivity has been observed in nanostructured metal thin films in the THz range [22]. These results are in good agreement with the predictions of the modified Drude–Smith theory [23], which takes into account the environment dictated asymmetry of the carrier scattering in a thin film.

Motivated by the idea of proposing a simple versatile tool for the design and fabrication of graphene-enhanced passive devices based on gold-plated patterned dielectric substrates, in this work, we investigate the influence of the non-homogeneity and roughness of the gold thin film, mostly originating from the surface imperfection of the SiO_2_ lattice, on the THz performance of the metasurface. Further, we demonstrate that the use of the Drude–Smith model for gold conductivity allows introducing into the simulations the experimentally observed oscillations of the THz response.

## 2. Materials and Methods

### 2.1. Metasurface Fabrication

The THz grating was fabricated by femtosecond micromachining of the fused silica SiO_2_ substrate (Casix Inc., Fuzhou, China). In the experiments, laser pulses at the central wavelength of λ = 792 nm, pulse duration of 150 fs, and repetition rate of 1 kHz were employed. The pulse energy was fixed at 1.25 mJ. The Gaussian beam of 7.5 mm in diameter was focused on the surface of a substrate using an F = 20 mm focusing lens (Thorlabs, Newton, MA, USA). The distance between the lens and the sample and the scanning speed of 0.5 mm/s were set to fabricate grooves with a width of w = 100 μm and a depth of h = 80 μm. At such parameters, we fabricated a periodic grooves structure with a period of p = 167 µm. The d_g_ = 30 nm gold conductive layer was deposited on the micromachined substrate using Emitech K675X sputter (EMITECH, Ashford, UK) at the current of 350 mA in Argon plasma (Linde Oy, Espoo, Finland). The next step was to transfer the t_pb_ = 500 nm thick polymethylmethacrylate (PMMA) spin-coated layer (Allresist GmbH, Strausberg, Germany) onto the patterned substrate. PMMA was spin-coated at 1800 rpm on the Cu foil (Alfa Aesar, Massachusetts, MA, USA), and baked at 150 degrees Celsius for 5 min. The next step is Cu foil etching in FeCl (Sigma-Aldrich, St. Louis, MA, USA), washing PMMA film in distilled water, and transferring to the prepared patterned SiO_2_ substrate covered with gold (Sigma-Aldrich, St. Louis, MA, USA). To improve adhesion, we first cleaned the patterned substrate in acetone and isopropanol, dried it with nitrogen gas, and then finally ran 30 s oxygen plasma (Plasma Etch, Carson City, NV, USA). Standard manual technology of ‘fishing out’ the polymer film onto a substrate was used along with further drying it overnight and then baking it on a hotplate at 60 °C for 10 min to further improve the adhesion of the polymer thin layer to the metasurface by removing any residual water molecules. This layer was used to prevent electrical contact between the gold layer and the graphene sheet.

Graphene was grown on Cu foil in a conventional CVD methane-based process. Methane-based graphene CVD begins with the nucleation of the individual grains dispersed randomly over the copper surface. As time goes on, these grains grow continuously until they integrate and fuse in the end to form a continuous crystalline film [24]. Graphene was synthesized using a temperature of 1000 °C, for a dwell time of 1 h, with methane being introduced for the last 30 min of this dwell time. The graphene was spin-coated with PMMA polymer (t_ps_ < 150 nm, transparent for THz radiation), used for its handling.

**Figure 1:**
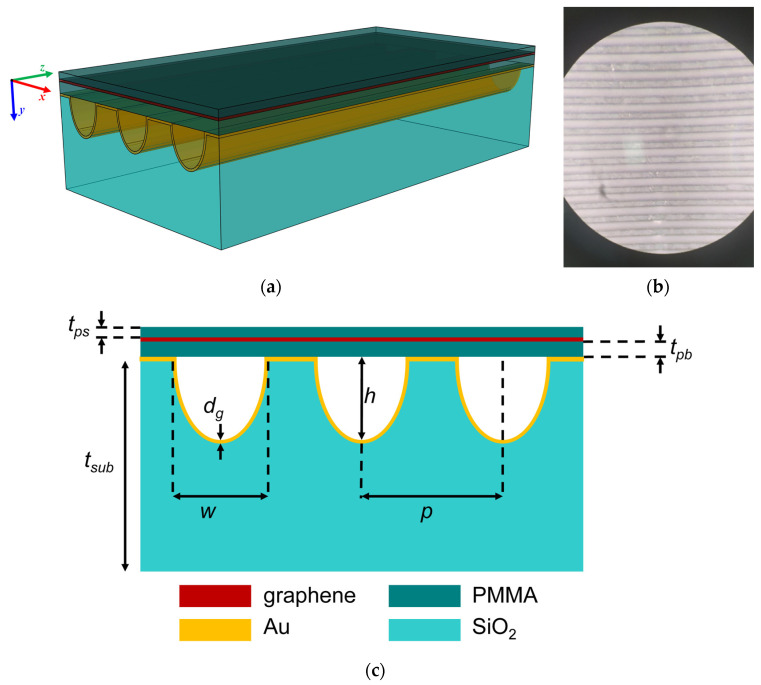
(**a**) Three-dimensional sketch of the graphene/gold/patterned SiO_2_ metasurface. (**b**) Photo of the top view of the graphene/gold/SiO_2_ patterned metasurface. (**c**) Cross-section of the metasurface.

The sample was then baked for 10 min at 60 °C. To remove the back graphene from the other side of the Cu foil, oxygen plasma treatment was employed. FeCl_3_ (Sigma-Aldrich, St Louis, MA, USA) was used as an etchant to remove Cu foil from the synthesized graphene covered with PMMA. The sample was left overnight in the etching solution for better results. Deionized water was used to remove residual FeCl_3_ solution by transferring it from the etching solution to a beaker filled with deionized water for 1 h. Using microscopic slide glass, the sample was then transferred onto PMMA film supported by the gold-plated patterned SiO_2_ grating. The metasurface 3D sketch (not to scale) is shown in Figure 1a, the photo showing the sample top view is presented in Figure 1b, and the metasurface cross-section is displayed in Figure 1c.

### 2.2. Metasurface Characterization Techniques

Structural characterization of the metasurface was performed by Zeiss SEM-LEO 1550 Gemini (Zeiss, Jena, Germany) and AFM Certus Light V (NanoScan Technologies, Dolgoprudny, Russia). Raman spectrum of graphene was recorded with HORIBA XploRA PLUS System (HORIBA, Palaiseau, France) at room temperature with a 1200 lines/mm grating. The measurements were performed using excitation at the wavelength of 532 nm and power below 0.8 mW. A 100× objective with NA = 0.95 was used. The sport size on the sample surface was 0.75 µm.

The THz transmission and reflection spectra were collected with a THz time domain spectrometer T-SPEC Ekspla (Ekspla, Vilnius, Lithuania). A 1050 nm pumping laser with 100 fs pulse duration at output power > 40 mW and pulse repetition rate of 80 MHz was used to generate THz radiation. All measurements were performed with a normally incident THz radiation and the electric field was oriented with the grooves. The measurement details can be found in [12]. For 2D transmission imaging, the XY two-dimensional motor stage was assembled into the system. Images were recorded by scanning the sample across the focus of the THz beam. The maximum scanning range of the two-dimensional motor stage is 20 mm × 20 mm. The diameter of the beam in focus point is around 2 mm.

### 2.3. Metasurface FEM Modeling

**Figure 2:**
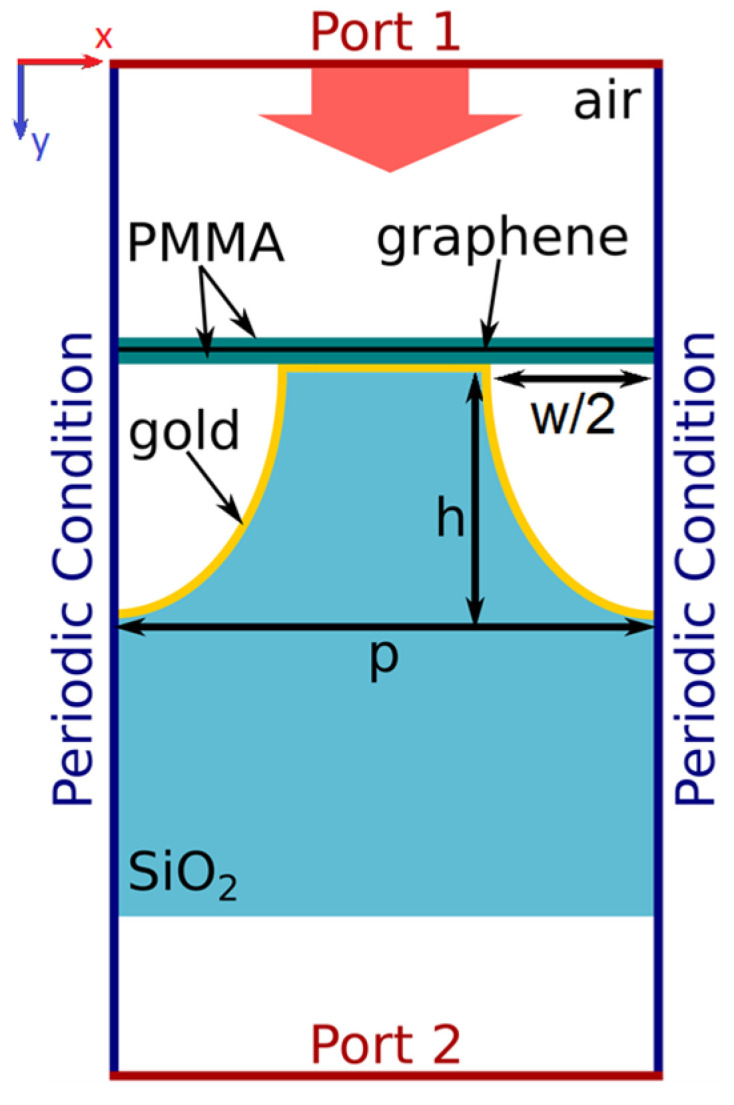
The graphene/gold/patterned SiO_2_ metasurface FEM model under THz irradiation in COMSOL.

The fabricated structure was modelled using the commercial FEM software. COMSOL Multiphysics^®^ v.5.3a (COMSOL Inc., Burlington, MA, USA). The structure can be assumed to be infinite in the longitudinal direction; thus, the problem can be reduced to a 2D model. The distance from both ports to the metasurface is much longer than the wavelength. The FEM model is shown in Figure 2.

**Figure 3:**
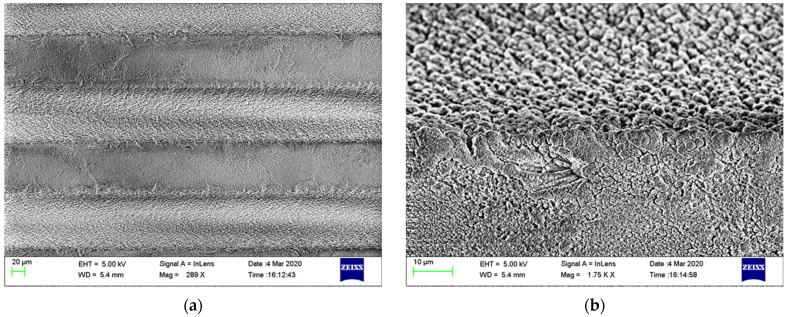

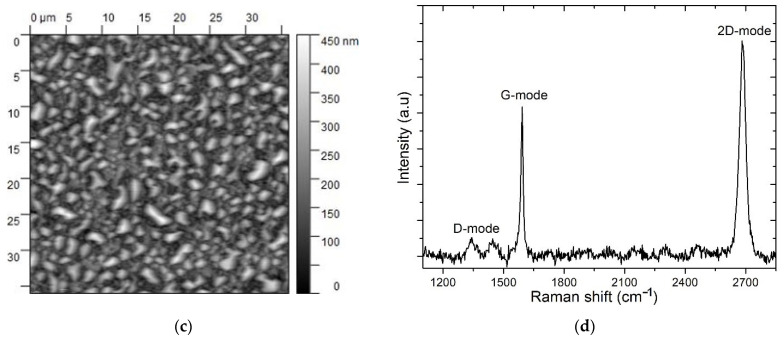
(**a**,**b**) SEM images of patterned SiO_2_/gold diffraction grating, where (**b**) demonstrates the groove interface. (**c**) AFM of the plane SiO_2_ surface covered with gold. (**d**) Raman spectra of CVD graphene used for metasurface fabrication.

In the plane wave approximation, we used periodic boundary conditions to replicate one spatial period and took into account the ellipsoidal shapes for the substrate grooves (see Figure 3 for SEM images at different magnifications). Table 1 summarizes the geometrical characteristics of the THz grating we used in the FEM model.

The relative permittivity of the SiO_2_ substrate and PMMA layers is set to ε_s_ = 3.8 [25] and ε_p_ = 2.6 [26], respectively. According to the literature data, both materials have insignificant absorption in the considered frequency range ([25,26] for SiO_2_ and PMMA, respectively); therefore, their loss tangent is set to zero.

#### 2.3.1. PMMA/Graphene/PMMA Layer

In the terahertz and microwave ranges, the graphene surface conductivity σ_gr_ is mainly determined by intraband transitions. In the numerical simulation, we employed the following equation for graphene surface conductivity [15]:

(1) $$\sigma_{\mathrm{gr}}\left(\omega ,\mu ,T,\gamma_{\mathrm{trans}}\right)=\frac{2e^{2}k_{B}T}{\pi \hbar^{2}}\ln\left(2\cos h\left(\frac{\mu }{2k_{B}T}\right)\right)\frac{i}{\omega +{i\gamma }_{\mathrm{trans}}}$$

where μ = 0.1 eV (unless other stated) is the graphene chemical potential, T is the room temperature, γ_trans_ *=* 13 THz, e is the electron charge, $k_{B}\;$ is the Boltzmann constant, *ℏ* is the reduced Plank constant, ω is the angular frequency, and i is the imaginary unit. The EM response of the graphene sheet was described by introducing the electrical current density at the plane interface between the two PMMA layers, where currents in the x and z directions are functions of the graphene surface conductivity (1) and of the local electric field, while the y-component of the electrical current (normal to the graphene surface) is zero.

#### 2.3.2. Gold Film

In order to achieve maximum reflectivity, the metal thickness must be at least twice the skin depth (δ) at the frequency of the incident THz radiation [27]. In the considered terahertz frequency range, the skin depth of gold is at least 25 nm [28,29]; therefore, the minimum gold thickness providing mirror-type reflection is higher than the thickness of the gold layer in the considered device. That is, for the percolated, but not saturated system made of defected 30 nm thick gold, the condition of achieving perfect reflection is far from being satisfied [30].

Bulk metals can be well described in terms of the Drude model, which predicts very weak frequency dependence of the complex conductivity in the THz range. This is not necessarily correct for thin metal films. Their inhomogeneity, caused by the roughness of the substrate surface, together with the roughness and graininess of the gold, may result in a significant deviation from the classical Drude-like behaviour, especially in the case of layers thinner than the skin depth. Additional electron scattering mechanisms due to impurities lead to a significant decrease in its conductivity compared with bulk [31]. As a result, an effective conductivity is used in the model to describe the experimental response, which takes into account the peculiarities of the material. Electromagnetic modeling of the conducting films with a thickness significantly smaller than the wavelength can be performed by means of the surface conductivity [16,29,31,32]. In the case of ultrathin metal films, the phenomenological extension of the Drude model, the Drude–Smith model [29], which takes into account the scattering of the charge carriers, was successfully adopted for different nanosystems and a wide variety of materials [33]. The alternative method for the case of the grained films is the percolation theory [34], which provides a good agreement for the far-IR region, but is more complex to apply owing to the involvement of specific scaling functions, which are determined by the microgeometry of a film [22]. Another method that can be applied for defective films is the effective medium theory [35]. However, as highlighted in [29], such media is not considered to be in contact with any interface and, as a result, interaction with the dielectric substrate is usually neglected. Meanwhile, the Drude–Smith approach shows good agreement between theory and experiment in the case of a dielectric substrate supporting a thin (comparable to the mean free path of the charge carrier) metal film with grains [22,29]. As demonstrated below, it accomplishes our aim of highlighting the influence of the defective thin gold film, allowing us to model the studied system with sufficient precision.

In the fabricated metasurface, the gold layer thickness is much smaller than the wavelength; thus, in the FEM model, it can be described by introducing the surface current density in boundary conditions, i.e., the value of the current density on the surface, according to

(2) $$\;\underset{-}{J}\;=\sigma_{g}\;\underset{-}{E}\;$$

where σ_g_ is the effective sheet conductivity of the gold. In the framework of the Drude–Smith model [22,29],

(3) $$\sigma_{g}=d_{g}\frac{\varepsilon_{0}{\tau \omega }_{p}^{2}}{1-i2\pi f\tau }\;\left(1+\frac{c}{1-i2\pi f\tau }\right)$$

where ε_0_ is the vacuum permittivity, τ is the electron momentum relaxation time, ω_p_ is the plasma frequency, and f is the frequency. The gold layer thickness d_g_ was added in the original formula for conductivity, transforming the volume conductivity into the sheet one. As it was impossible to measure d_g_ in the experiment, it can be used as a parameter in the model. As the thickness of the layer is smaller than the gold skin depth, a linear dependence for the thickness was expected. The second term in the brackets in Equation (3) represents the effect of the persistence of the conduction electron’s initial velocity only during the first collision, following the assumption that the electron fully lost its momentum after the first scattering event. The constant c is c = <cos θ> [23], where θ is the scattering angle, and the angular brackets stand for statistical averaging. As cos θ is negative when the momentum of the carrier reverses, negative c indicates that backscattering dominates. At c = −1, all scatters revert to the propagation direction after scattering. At c = 0, the scattering is isotropic, i.e., carriers are equally scattered in all possible directions. In such a case, the Drude–Smith model reduces to the classical Drude one.

#### 2.3.3. Input/Output of the Numerical Simulation

The top and bottom input/output ports are placed above the graphene sheet and below the silica substrate (see Figure 2). By exciting one of the two ports, we can obtain the metasurface THz transmission, reflection, and absorption coefficients for the irradiation of the metasurface from the graphene and substrate sides.

The FEM numerical simulation allows us to reveal how the THz performance depends on the metasurface geometry, providing the guidelines for the design of the best-performing configuration and/or the most robust. In this work, the simulation was performed to estimate the impact of the gold reflector layer conductivity on the overall device performance.

In agreement with the experimental procedure, the top port was powered to simulate the radiation propagating from a source located on the top of the patterned surface. The electric field was set parallel to the grooves’ orientation, i.e., the electric field is orientated along z and the direction of propagation is y.

## 3. Results

### 3.1. Metasurface Characterization

#### 3.1.1. Structure Characterization

Structural characterization by SEM (see Figure 3a,b) shows that the surface of the grooves is not perfect. Both the top and bottom parts of the patterned SiO_2_ grating have a porous surface with noticeable roughness at the level of 100 nm–1 μm. Being at least 300 times smaller than the wavelength, this roughness does not influence the THz response of the metasurface. However, it will affect the homogeneity and even integrity of the gold layer deposited on the grating surface.

As one can see from the AFM images taken from the plane part of SiO_2_ covered with gold (Figure 3), the gold covered surface has granules of 1–5 μm in lateral dimensions, whereas the remaining part of the sample plane surface is covered by homogeneously distributed golden domains much thinner than the granules’ height.

The Raman spectrum of CVD graphene used for metasurface fabrication is presented in Figure 3d. One can see the three most important spectral features typical for graphitic materials, that is, the D-band at ~1341 cm^−1^, the G-band at ~1590 cm^−1^, and the 2D band at ~2685 cm^−1^. The 2D-mode exhibits a sharp (full width at half maximum, FWHM, ~35 cm^−1^) peak, which is 1.5 times more intense than the peak of the G-mode, indicating the good quality of the synthesized graphene.

#### 3.1.2. Terahertz Probing

**Figure 4:**
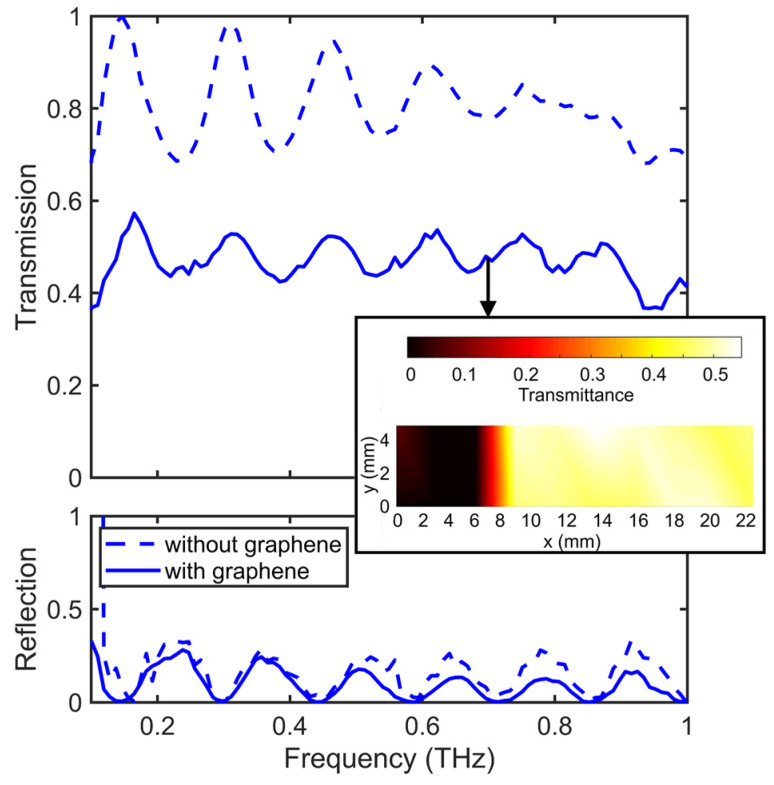
THz spectra of metasurface graphene/gold layer on patterned SiO_2_, measured when radiation comes from the graphene side. Inset: the THz-TDS transmission image (plotted at 0.7 THz) of the groove’s border. The size of the scanned area was 22 mm × 5 mm. The step size was set at 2.5 mm in the horizontal direction.

The measured transmission and reflection spectra for the metasurface with and without the PMMA/graphene/PMMA sandwich structure are presented in Figure 4. The point-by-point THz spectral imaging at 0.7 THz of the 22 mm × 5 mm scanned area, which includes the grating structure and untreated part, is presented in the inset of Figure 4. As can be seen, the gold conductive layer deposited on the flat substrate works as a perfect reflector (T = 0, R = 1). At the same time, the micromachining of SiO_2_ leads to the formation of the macroscopic features in the patterned area, which, in addition to the imperfectness of the deposited gold layer, results in a decrease in the overall effective conductivity of the conductive layer and increase in the total transmission. Hence, the transmission and reflection of the PMMA/graphene/PMMA deposited on the grating structure vary in the vicinity of 50% and 10%, respectively. Thus, the measured absorption A = 1 – T − R of the metasurface is close to 40%, which, as one can see, is mainly determined by the PMMA/graphene/PMMA layer.

### 3.2. Evaluation of the Gold Thin Film Conductivity Effect by the FEM Model

**Figure 5:**
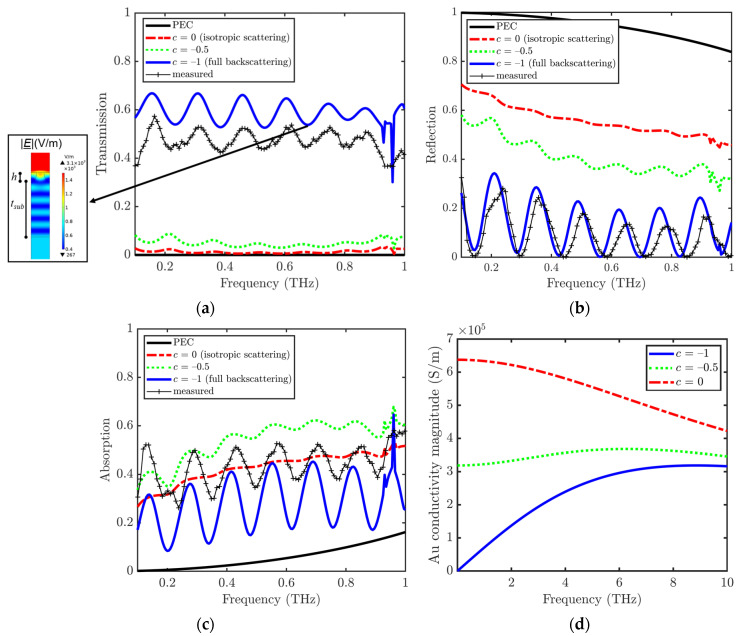
Simulated power (**a**) transmission, (**b**) reflection, and (**c**) absorption spectra of the graphene/gold/patterned SiO_2_ metastructure computed by FEM simulations, at different c corresponding to different conditions of gold nonideality, exciting the top port with a THz electric field parallel to the grooves and normally incident on the device top surface, in the frequency range of 0.1–1 THz. (**d**) The magnitude of the gold layer complex conductivity in the frequency range 0.1–1 THz. Inset of (**a**): electric field magnitude distribution in the cross-section surface at f = 0.685 THz, in correspondence with a minimum of T and R and a maximum of A. In order to evidence the grooves’ location, h and t_pb_ parameters are also reported here.

We performed a comparative study of the metasurfaces based on the perfect conductor (PEC, σ_g_ → ∞), Drude metal (c = 0 in Equation (3)), and Drude–Smith metal with dominating backscattering [23] of carriers (c < 0 in Equation (3)) by means of simulations of the FEM model described in Section 2.3, when the top port transmits a THz wave normally incident on the top surface, with the electric field parallel to the orientation of the grooves. As can be seen in Figure 5, the non-ideal conducting behavior of gold greatly affects the power transmission and reflection shown in Figure 5a,b, respectively. The inset of Figure 5a reports the electric field distribution in the grooves and the substrate at f = 0.685 THz for the case c = −1, in correspondence with a minimum of R and T and a maximum of A. Concerning the curves in Figure 5a–c, it can be noticed that an ideal behaviour of the gold reflector (PEC layer) would prevent any transmission to the substrate, thus avoiding any evidence of the Fabry–Pérot fringes. In this case, T = 0 and A = 1 − R. For the Drude metal layer (c = 0), the transmittance is low, and weak interference fringes can be seen for T, R, and A, caused by the multiple reflections at the interfaces with the substrate. For the Drude–Smith layer at c = −0.5 (carriers are equally scattered backwards and towards the substrate), T increases and *R* decreases with respect to those of the Drude metal. Finally, for the Drude–Smith layer at c = −1 (backscattering dominates), the transmission is much higher than that for the Drude metal. This phenomenon can be explained by taking into account that, at c = −1, the material behaves like an insulator. That is, in such a case, the backscattering prevents the propagation of electrons when a static electric field is applied, i.e., σ_g_ = 0 at f = 0 in Equation (3) [29]. The full backscattering condition causes the increase in the Fabry–Pérot fringes.

One can observe from Figure 5d that, the stronger the backscattering, the lower the complex conductivity magnitude. This behavior has been frequently observed in nanostructured metal films that behave similarly to poor conductors in the proximity of the metal-insulator percolation transition owing to the increased scattering on grain boundaries and other defects [31,36].

By comparing the simulation results with the experiments in Figure 5, it can be concluded that a value of c between −0.5 and −1 corresponds to the observed THz behavior of the graphene/gold/patterned SiO_2_ metasurface. In particular, the value of c that better allows reproducing the experimental result is very close to −1. Although the simulation results do not match perfectly the experimental data, they well reproduce the qualitative features of the spectrum, including the number of oscillations and the amplitude envelope. This demonstrates the efficiency of the proposed approach.

## 4. Conclusions and Future Work

In this work, we studied CVD graphene-based metasurfaces for THz radiation. The SEM and AFM images show that the femtosecond micromachining results in the rough and porous surface of the patterned SiO_2_. This may lead to the significant overestimation of the Au thickness and its sheet conductivity because the effective surface area of the patterned substrate is greater than that of the flat one.

In order to reveal the impact of the gold layer imperfectness on the metasurface THz performance, we employed FEM numerical simulation by taking into account the asymmetry of the carriers scattering in the thin metal film. Using the Drude–Smith model to describe the gold film ac conductivity, we compared its predictions with those given by the perfect conductor and the Drude models.

The obtained results show that the Drude–Smith model is capable of describing the measured spectra of the transmission, absorption, and reflection of the SiO_2_ grating covered by the deposited thin gold film. In particular, the developed model well describes the Fabry–Pérot interference pattern, as well as other EM features inherent for the experimental behavior of the metasurface, enabling future investigation of the metasurface performance in different applications. Our experimental findings confirm that the predominant backscattering of the carriers in the thin film does affect the THz properties of the gold layer and strongly influences the performance in the frequency range of interest. One may expect that THz performance of the metasurface can be substantially improved if an alternative physical deposition technique (e.g., thermal vacuum deposition, electroplating, and so on) is used.

## Figures and Tables

**Table 1:** The metasurface geometrical parameters.

Symbol	Quantity	Value
Vertical dimensions		
t_sub_	SiO_2_ substrate	500 μm
t_pb_	PMMA buffer layer	500 nm
t_ps_	PMMA support layer	150 nm
d_g_	Gold film thickness	30 nm
h	Groove height	80 μm
Lateral dimensions		
p	Spatial period	167 μm
w	Groove width	100 μm

## Data Availability

Not applicable.
